# Integrated Ultra-Wideband Microwave System to Measure Composition Ratio Between Fat and Muscle in Multi-Species Tissue Types

**DOI:** 10.3390/s25175547

**Published:** 2025-09-05

**Authors:** Lixiao Zhou, Van Doi Truong, Jonghun Yoon

**Affiliations:** 1 Department of Mechanical Design Engineering, Hanyang University, 222, Wangsimni-ro, Seongdong-gu, Seoul 04763, Republic of Korea; 2 Department of Mechanical Engineering, Hanyang University, Ansan 15588, Republic of Korea; 3 AIDICOME Inc., Ansan 15588, Republic of Korea

**Keywords:** ultra-wideband microwave, non-destructive testing, tissue composition prediction, random forest regression, fat thickness, muscle thickness

## Abstract

Accurate and non-invasive assessment of fat and muscle composition is crucial for biomedical monitoring to track health conditions in humans and pets, as well as for classifying meats in the meat industry. This study introduces a cost-effective, multifunctional ultra-wideband microwave system operating from 2.4 to 4.4 GHz, designed for rapid and non-destructive quantification of fat thickness, muscle thickness, and fat-to-muscle ratio in diverse ex vivo samples, including pork, beef, and oil–water mixtures. The compact handheld device integrates essential RF components such as a frequency synthesizer, directional coupler, logarithmic power detector, and a dual-polarized Vivaldi antenna. Bluetooth telemetry enables seamless real-time data transmission to mobile- or PC-based platforms, with each measurement completed in a few seconds. To enhance signal quality, a two-stage denoising pipeline combining low-pass filtering and Savitzky–Golay smoothing was applied, effectively suppressing noise while preserving key spectral features. Using a random forest regression model trained on resonance frequency and signal-loss features, the system demonstrates high predictive performance even under limited sample conditions. Correlation coefficients for fat thickness, muscle thickness, and fat-to-muscle ratio consistently exceeded 0.90 across all sample types, while mean absolute errors remained below 3.5 mm. The highest prediction accuracy was achieved in homogeneous oil–water samples, whereas biologically complex tissues like pork and beef introduced greater variability, particularly in muscle-related measurements. The proposed microwave system is highlighted as a highly portable and time-efficient solution, with measurements completed within seconds. Its low cost, ability to analyze multiple tissue types using a single device, and non-invasive nature without the need for sample pre-treatment or anesthesia make it well suited for applications in agri-food quality control, point-of-care diagnostics, and broader biomedical fields.

## 1. Introduction

Body composition is the result of complex interactions among various biological tissues and components, which influence physiological processes and pathological mechanisms in specific ways [1]. Accurate and non-destructive assessment of body composition—particularly fat, moisture, and lean tissue content—is becoming increasingly important in numerous fields, including food quality control, biomedical modeling, and the development of portable sensing systems [2,3]. Body composition refers to the proportion of different tissues and substances in the body, including adipose tissue, water, lean tissue (primarily composed of muscle, bone, and internal organs), and minerals. Among these components, fat content and its distribution exhibit the highest variability in animal carcasses. Fat content is negatively correlated with lean meat yield, and consequently, with the commercial value of the carcass [4]. Carcasses with excessive subcutaneous fat are often penalized by meat processors, as the increase in fat content is inversely related to the amount of marketable lean meat, thereby reducing the overall carcass value [5]. Furthermore, due to consumer preferences for meat with lower subcutaneous fat content [6], excessive fat levels increase labor costs during processing to meet market requirements. These factors highlight the necessity for precise and objective assessment of meat composition.

In addition to applications in food science, the assessment of body composition is critically important for human health. A reduction in muscle mass has been linked to numerous adverse outcomes, including increased risk of postoperative complications, diminished physical function, reduced quality of life, and shorter lifespan [7,8,9]. This is especially significant in elderly populations, where the decline in muscle quality—known as sarcopenia—can significantly impair mobility, increase the risk of falls and morbidity, and shorten life expectancy [8,9,10]. Sarcopenia is now recognized as a key medical risk factor for both mortality and disease burden. It is estimated that the number of people with sarcopenia in Europe will rise from 19.7 million in 2016 to over 32 million by 2045 [11].

Meanwhile, uncontrolled fat accumulation also poses significant health risks [12]. The widespread adoption of high-calorie diets and sedentary lifestyles has made obesity and type 2 diabetes major contributors to coronary artery disease progression and mortality [13]. Large-scale studies, such as the Framingham Heart Study and the Nurses’ Health Study, have shown that obese individuals face twice the risk of heart failure and a 4.1-fold increased risk of cardiovascular disease progression compared to individuals with normal body weight [14,15].

Taken together, these findings underscore the urgent need for reliable and non-invasive methods to detect and quantify body composition in both meat products and the human body. Developing technologies capable of addressing this need is essential for improving public health, optimizing food processing, and advancing biomedical research.

To detect body composition in meat and the human body, several existing methods have been developed, yet each presents certain limitations. Traditional techniques for meat composition analysis—such as chemical extraction, near-infrared spectroscopy (NIR), and imaging-based approaches—have been widely used but present significant limitations for real-time, non-destructive applications [12,16]. Chemical extraction provides relatively accurate quantification by isolating specific components through solvent-based processes. However, it is inherently destructive, labor-intensive, and time-consuming [17]. The requirement for specialized laboratory equipment and trained personnel further limits its suitability for rapid or field-based assessments [18].

Near-infrared spectroscopy (NIR) offers a non-invasive and rapid alternative, relying on molecular absorption of near-infrared light to infer chemical content [19,20]. Although efficient, factors such as the physical state of the sample (minced or intact) [21], overlapping absorption peaks of different components (such as fat, moisture, and protein) [22,23], and the number of calibration samples [24,25] negatively affect the accuracy of NIR in composition detection. Advanced imaging techniques, such as ultrasound imaging, enable high-resolution and non-destructive tissue characterization. However, ultrasound measurements are susceptible to various factors, including the irregular shape of the meat, uneven distribution of fat and lean tissue, measurement location, and ultrasound frequency, all of which can lead to significant measurement errors [26]. Although these traditional methods provide valuable insights, their limitations in destructiveness, cost, portability, and efficiency underscore the need for alternative approaches.

In parallel, human body composition is typically assessed using clinical tools such as dual-energy X-ray absorptiometry (DXA) and bioelectrical impedance analysis (BIA). DXA is considered a gold-standard method for quantifying bone mineral content, fat mass, and lean mass, offering high accuracy and repeatability [27]. However, it requires expensive, non-portable equipment and involves exposure to low-dose radiation, making it unsuitable for frequent or widespread use outside clinical settings [28,29,30]. On the other hand, BIA estimates body composition by measuring the resistance of body tissues to a small electrical current [31]. While BIA is inexpensive, portable, and easy to operate, its accuracy can be significantly affected by factors such as hydration status, electrode placement, and individual variability in tissue conductivity [32,33].

These limitations significantly reduce the reliability of BIA in specific populations, including Asian individuals, due to the reliance on equations developed primarily for Western populations and the influence of environmental factors such as temperature and humidity. Studies have shown that BIA accuracy in Asian children and adults is often lower compared to DXA, highlighting the need for population-specific calibration. Taking together, these challenges further highlight the demand for alternative, flexible, and non-invasive technologies that can deliver reliable body composition assessments across diverse settings.

Microwave sensing (MiS) technology has demonstrated significant potential across various fields, particularly in non-destructive testing and medical imaging. As electromagnetic waves operating in the frequency range of 300 MHz to 300 GHz, microwaves possess unique physical characteristics that make them highly suitable for such applications [34,35]. Due to their high sensitivity to dielectric properties, microwave signals can characterize biological tissues in a non-invasive manner [36]. Dielectric properties describe a material’s ability to store and dissipate electrical energy under an electric field, and in biological tissues, these properties are closely related to composition and structure. For instance, fat, water, and lean tissue exhibit markedly different dielectric constants—water having a higher value, while fat presents a relatively lower one. In human tissues, different components interact with microwaves differently: muscle tissue tends to absorb microwaves more strongly, whereas fat shows more pronounced scattering effects [37]. These contrasts can be detected by microwave sensors to distinguish tissue types and analyze their composition. In food analysis, microwaves can penetrate both packaging and the food itself, enabling rapid, non-destructive detection of water and fat content, thereby supporting efficient quality control.

In microwave sensing technology, the scattering parameter (S11) serves as a critical diagnostic metric, widely applied in biological tissue composition analysis and food quality control. This S11 reflects the reflection characteristics of electromagnetic waves at the material interface, and its variations are closely correlated with the dielectric properties of the tissue. Studies have shown that changes in fat layer thickness can lead to significant shifts in the S-parameter, indicating its potential for estimating subcutaneous fat thickness [38]. However, due to the multilayer structure of the human abdomen and the occurrence of total internal reflection, accurate measurement of the S11 becomes challenging [39]. To address this issue, machine learning algorithms can be introduced to learn from and process the S11 data, thereby improving both accuracy and analytical efficiency.

Recent studies have explored the integration of microwave reflection parameters with machine learning to estimate body composition indicators. Mattsson et al. [40] employed microwave data collected from 41 volunteers and constructed a three-stage regression pipeline comprising SelectKBest, support vector machines, AdaBoost, and random forest algorithms. Their approach aimed to predict ultrasound-measured skin thickness between 1.5 and 2.7 mm, fat thickness ranging from 6 to 15 mm, and muscle cross-sectional area from 1.7 to 8 square centimeters. The best predictive performance was observed in fat and muscle thickness, with coefficients of determination reaching 0.57 and 0.63, respectively, based on a hybrid dataset. However, the model was trained on only 35 valid cases, and the prediction accuracy for skin thickness was notably poor, with a coefficient of determination of minus 0.32. These findings raise concerns regarding the model’s stability and its generalizability to clinical settings.

Mattsson et al. [41] developed prediction models for CT-measured carcass fat, lean, and bone percentages using UWB microwave scans at the 12th/13th rib, located 45 mm from the spinous process, in a study involving 343 lambs. Ensemble stacking algorithms were applied using WEKA 3.9.4, and five-fold cross-validation yielded R^2^ values of 0.78 for fat, 0.64 for lean, and 0.75 for bone, with corresponding RMSEPs of 2.39%, 2.15%, and 0.99%. However, due to the limited number of samples within the extreme composition ranges—particularly high fat or low and high lean content—the prediction errors exceeded the AUS-MEAT allowable threshold of ±3%, emphasizing the need for expanded datasets to enhance model robustness at distribution boundaries.

Ghosh and Gupta [42] developed a four-layer neural network with two ReLU-activated hidden layers of 22 and 15 neurons, trained using the Adam optimizer and mean absolute error loss. The model was based on 110 CST-simulated samples to predict the performance of a 2.45 gigahertz muscle-implanted antenna, including reflection coefficient, realized gain, and fractional bandwidth. Simulations varied skin thickness from 0.05 to 4 mm and fat thickness from 1.6 to 15 mm. On ten unseen cases, the model achieved a mean absolute error below 0.99 percent. However, its applicability remains limited due to reliance on a single antenna design and a simplified three-layer phantom.

While previous studies on microwave-based tissue composition analysis have demonstrated potential, they often suffer from several key limitations. Most existing models have been developed and validated using specific types of biological samples—often under tightly controlled laboratory conditions—which limit their applicability to real-world scenarios. These models typically assume consistent tissue structure and homogeneous dielectric properties, making them less effective when applied to biological tissues with more complex or variable compositions. As a result, their ability to generalize across different tissue types, such as those with varying fat, muscle, and moisture content, remains limited. In particular, muscle-related predictions tend to exhibit only moderate accuracy, and performance often degrades near the extremes of composition distributions. Few efforts have succeeded in developing a compact, low-cost, and versatile device that can accurately assess diverse biological tissues using a unified system.

In this study, a compact and cost-effective microwave sensing (MiS) system was developed to experimentally predict composition ratios for different types of meat samples by analyzing the S11 reflection coefficient across a broad frequency spectrum. Our proposed solution overcomes above barriers by delivering robust and accurate predictions across pork, beef, and oil–water samples using a single handheld unit. The device seamlessly connects via Bluetooth and requires no additional hardware adaptation for different meat types, making it a highly practical tool for both agri-food inspection and biomedical sensing applications. The system capitalizes on the high sensitivity of microwave signals to the dielectric properties of biological tissues, with the S11 parameter serving as a critical indicator of tissue compositional variations. System performance was evaluated using three representative sample types—pork, beef, and oil–water mixtures—which exhibit marked differences in fat distribution and moisture content. This selection underscores the system’s capability to generalize across heterogeneous tissue types without necessitating hardware modifications or reconfiguration, thereby demonstrating its versatility for diverse practical applications. In practice, this system enables fast, non-invasive meat quality checks in food processing and offers potential for body composition analysis in healthcare and fitness. Its portability and versatility make it ideal for on-site, real-time measurements without complex equipment. The device’s portable and lightweight design facilitates deployment in diverse environments, including clinical settings and field-based food quality assessments, while its low production cost supports scalability and broad accessibility.

The system’s non-invasive nature is a key advantage, eliminating the need for sample pre-treatment or anesthesia, which simplifies the measurement process and enhances user-friendliness. This feature is particularly significant in clinical and field settings, where rapid and safe assessments are crucial. Additionally, the system operates without the need for ionizing radiation, ensuring a high safety profile for both users and samples. These attributes collectively enhance the system’s practicality and applicability across various domains.

Frequency-domain signals acquired by the MiS system were subjected to comprehensive feature extraction, followed by regression-based prediction of fat thickness, muscle thickness, and fat-to-muscle ratios using multiple machine learning algorithms—Ridge Regression, K-Nearest Neighbors, Random Forest, and Artificial Neural Networks. These models were selected to evaluate the system’s adaptability to high-dimensional, non-invasive signal data and were systematically compared in terms of predictive accuracy, robustness, and generalization capacity.

The experimental protocol progressed in a stepwise manner, beginning with controlled oil–water mixtures and advancing to biologically complex pork and beef samples. This progressive validation strategy confirmed the system’s accuracy and robustness under increasingly realistic and challenging conditions, with complete tissue composition analysis achievable within a few seconds. Notably, our study places a particular emphasis on the detection of different species, aiming to unify the measurement approach while ensuring high correlation between true and predicted values. This focus on cross-species applicability and accuracy sets our work apart from other studies, which often concentrate on a single species or tissue type. By demonstrating the system’s ability to generalize across diverse biological samples, we provide a more comprehensive and versatile solution for tissue composition analysis. This original contribution represents a significant advancement in the field, offering a novel approach that unifies the detection of multiple species while maintaining high accuracy and reliability.

Collectively, these results demonstrate the system’s potential as a rapid, reliable, and versatile tool for human body composition assessment and broader biomedical and agri-food applications. The system’s unique combination of portability, non-invasiveness, high safety profile, and adaptability to diverse tissue types sets it apart from existing methods. Unlike traditional invasive techniques or those requiring extensive sample preparation, our system offers a streamlined, user-friendly approach that can be deployed in various settings without compromising accuracy or safety. This unified and versatile solution for tissue analysis across multiple species represents a significant step forward in the field.

## 2. Materials and Methods

### 2.1. Description of Microwave Hardware

**Figure 1:**
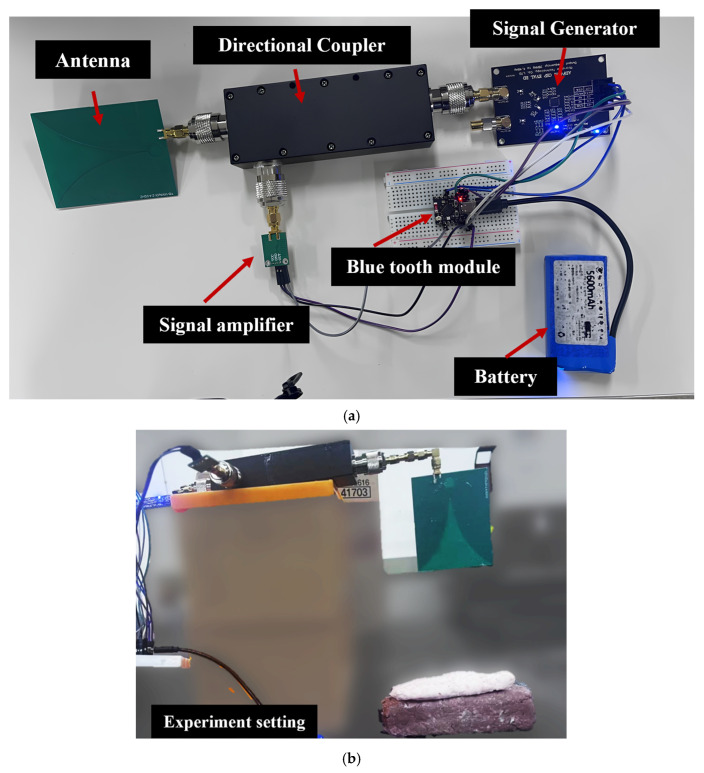
Microwave device measuring tissue depth with the center of Vivaldi patch antenna: (**a**) microwave measurement device components; (**b**) experimental setup showing antenna-to-sample positioning.

The development of a portable ultra-wideband microwave system, referred to as the Microwave System for In vivo Characterization (MiS), was presented in this study, specifically designed for the non-invasive characterization of biological tissues. The system is engineered as a standalone unit that integrates key functionalities of a vector network analyzer (VNA) along with a Bluetooth communication module. This integration significantly enhances the system’s portability and applicability in diverse real-world scenarios, offering a compact and practical solution for in-field measurements, as illustrated in Figure 1.

The MiS system comprises several critical components, including a Bluetooth module, a frequency synthesizer, a directional coupler, and an S11 signal detection unit. The compact and modular design of these components enable a high degree of integration, ensuring both operational efficiency and mechanical simplicity. To minimize electromagnetic interference and environmental noise, these sensitive components are enclosed within a metal shielding cover, effectively reducing signal disturbances and enhancing measurement stability. At the heart of the system lies the frequency synthesizer, which generates sweeping radio frequency (RF) signals within a predefined range of 2.4 GHz to 4.4 GHz. This range was carefully selected based on prior literature identifying 1–5 GHz as an effective band for tissue characterization, while also considering practical constraints such as data dimensionality, antenna size, and limitations in the signal generator’s performance. In particular, the lower bound of 2.4 GHz was chosen because the signal generator can only operate reliably above 35 MHz, which, combined with hardware and performance considerations, effectively determined the practical frequency range of the system [43,44].This frequency band was selected based on the dielectric response of biological tissues in this range, ensuring optimal sensitivity to variations in tissue properties. The generated RF signal is transmitted to the target sample via a compact and efficient Vivaldi antenna. Building upon previous studies on tissue thickness detection in lamb samples, the antenna features a linearly polarized configuration, enabling effective transmission and reception through a single module [45]. Operating over a frequency range of 2.4 GHz to 5 GHz, the Vivaldi antenna has a physical dimension of 84 mm × 65 mm × 1 mm and provides a gain of 8 dB. Its tapered-slot design ensures broadband operation, efficient energy transfer, and stable signal propagation. The compact and lightweight structure further enhances the system’s portability and practical deployment, making it well-suited for rapid, non-invasive tissue composition measurements.

**Figure 2:**
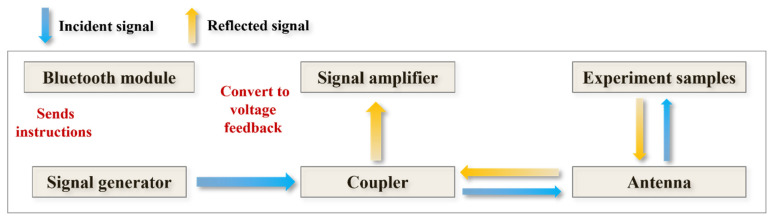
Schematic diagram of RF signal flow within the device.

Upon interaction with biological tissue, part of the RF signal is absorbed while the remainder is reflected. To accurately capture the reflected component, the system incorporates a directional coupler that separates the incident and reflected signals. This separation is crucial for precise measurement of the reflection characteristics, especially the reflection coefficient S11. By isolating the reflected signal, the system can reliably extract information regarding the dielectric properties of the sample. This mechanism enhances the precision of the measurement process and provides a robust foundation for downstream signal processing and analysis. The signal flow is illustrated in the schematic diagram, as shown in Figure 2.

The reflection coefficient S11 serves as a key indicator of the dielectric behavior of biological tissues. It reflects the extent to which the sample impedes the RF signal, a phenomenon governed by intrinsic electromagnetic parameters such as permittivity (ε), conductivity (σ), and permeability (μ) [46]. Variations in these parameters among different biological tissues lead to distinct impedance mismatches at tissue interfaces, resulting in unique reflection patterns. Accurate S11 measurements, therefore, offer valuable insights into tissue composition and structure, supporting reliable non-invasive characterization.

Building on this principle, the present study integrates and compares feedback signals under various experimental conditions. Common features were extracted across samples, and machine learning models were trained to relate these features to the underlying tissue properties. For meat samples, particular emphasis was placed on predicting fat thickness, muscle thickness, and fat-to-muscle ratios—parameters of high relevance in clinical and food quality contexts. By unifying both hardware and algorithmic components into a cohesive system, MiS is able to deliver real-time predictions with high reliability and accuracy.

**Figure 3:**
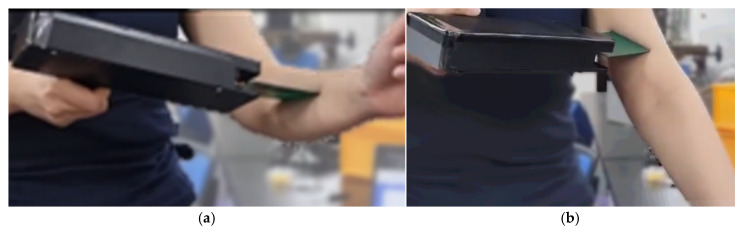
Handheld implementation of the MiS system for tissue measurement at the arm site: (**a**) Practical demonstration of handheld operation on the arm during tissue measurement; (**b**) Practical demonstration of handheld operation on the upper arm during tissue measurement.

To convert the power of the reflected wave into a measurable electrical signal, a logarithmic power detector was employed in the MiS system. The output voltage $V_{out}$, which corresponds to the reflected power level $P_{r}$, was generated and subsequently digitized by the microcontroller for further processing. This voltage response can be mathematically approximated by the following logarithmic relationship:

(1) $$V_{out}=A\cdot \log_{10}\left(P_{r}\right)+B$$

where A and B are calibration constants determined through system characterization. This relationship enables efficient transformation of the microwave reflection signal into an analog voltage domain, which is then digitized and wirelessly transmitted for further analysis. To facilitate convenient handheld operation following experimental measurements, and to enhance portability during device handling and transport, the entire system was enclosed within a lightweight ABS housing. This design not only protects internal components but also ensures ease of use in both laboratory and field settings. The finalized enclosure structure is illustrated in Figure 3.

### 2.2. Experimental Setup

**Figure 4:**
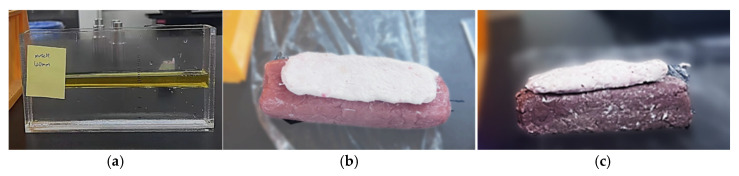
Microwave measurement configuration using a Vivaldi patch antenna for tissue-mimicking samples: (**a**) a mixture of flaxseed oil and deionized water; (**b**) minced pork tissue; (**c**) minced beef tissue.

To comprehensively investigate the microwave reflection characteristics of various biological and fluidic materials, this study meticulously prepared three distinct types of exes vivo samples encompassing a wide range of fat and muscle compositions, as shown in Figure 4. These samples include: (a) a mixture of flaxseed oil and deionized water; (b) minced pork tissue; and (c) minced beef tissue. The selection of these materials was strategically designed to represent different tissue types and enable a thorough analysis of the signal behavior associated with each.

The rationale behind selecting these three sample types lies in their ability to collectively simulate both simplified and structurally complex biological conditions. This allows the study to not only validate the sensing system under idealized dielectric conditions but also assess its performance in scenarios closer to real biological tissues.

For the first sample type, the combination of flaxseed oil and deionized water was chosen based on their well-characterized and contrasting dielectric properties. Previous studies have demonstrated that the dielectric constants of these two substances closely resemble those of human fat and muscle tissues, respectively [47]. Therefore, this mixture serves as an ideal surrogate model for simulating the dielectric behavior of human biological tissues. During preparation, the oil and water were carefully mixed at known volume ratios and manually layered to form a stratified structure. This configuration enabled controlled experimentation on how varying oil–water ratios influence the microwave reflection response.

To extend the analysis to more realistic biological media, meat-based samples were also incorporated. These samples introduce inherent dielectric complexity and structural heterogeneity, providing a rigorous testbed for evaluating the robustness of microwave signal interpretation. For the meat-based samples, pork and beef were selected due to their distinct fat and muscle compositions, which naturally exhibit differing dielectric characteristics. These differences represented the diversity found in biological tissues and enhanced the generalizability of the MiS system in detecting tissue composition across multiple species. In the sample preparation process, the intercostal muscles of pork and beef were used as muscle components, while fat tissues were harvested from the same species. All tissues were minced using a meat grinder to ensure homogeneity and consistency. Each sample was then manually arranged into a two-layer structure, with the fat layer uniformly distributed over the muscle layer. This arrangement was specifically designed to replicate the layered nature of biological tissue, allowing microwave signals to interact with the sample in a manner similar to in vivo conditions. The uniformity and flatness of each layer were carefully maintained to create a stable and repeatable measurement environment.

To ensure systematic data acquisition across a wide range of configurations, a well-defined parameter adjustment strategy was implemented. During sample fabrication, the thickness of the fat layer (or oil layer) was systematically varied from 5 mm to 29 mm in 3 mm increments, while the thickness of the muscle layer (or water layer) was adjusted from 25 mm to 40 mm in 5 mm increments. These step sizes were selected to generate a detailed and high-resolution dataset, enabling the capture of subtle variations in microwave reflection behavior across different tissue thicknesses. For each configuration, the mass of individual tissue components was accurately recorded, and the fat-to-muscle ratio was calculated. These parameters—including tissue thickness and composition ratio—served as the ground truth for model training and validation, providing a reliable foundation for analyzing the relationship between tissue structure and microwave reflectivity.

Microwave reflection measurements were performed using the portable MiS system described in Section 2.1. To ensure accuracy and stability during the measurements, a single antenna was fixed at a specific height to prevent movement or angular misalignment relative to the target surface. This positioning was essential for maintaining consistent signal coupling and minimizing variations due to mechanical instability. A direct-contact measurement approach was adopted to reduce signal attenuation and electromagnetic interference, thereby improving the accuracy and reliability of the measured reflection coefficient S11.

The system operates over a frequency range of 2.4 GHz to 4.4 GHz. Through repeated validation and experimental testing, this specific sub-band was confirmed to provide an optimal balance between resolution and system feasibility for our application.

Once the frequency sweep signal is emitted across the designated band, the reflected signal from the tissue surface is captured and analyzed to extract the reflection coefficient S11, which quantifies the proportion of energy reflected back toward the sensor. The reflected signal is first converted into an analog voltage via a logarithmic power detector. This analog signal is then digitized by a microcontroller unit (MCU), ensuring compatibility with digital processing frameworks.

To facilitate flexible and real-time data acquisition, the digitized signal is transmitted wirelessly via a Bluetooth module to an external host device. On the receiving end, custom-designed software reconstructs the corresponding S11 values from the transmitted data. This wireless communication scheme enables efficient signal analysis and reliable data collection under varying sample configurations, making the system suitable for portable and non-invasive diagnostic settings.

### 2.3. Description of MiS Signal Processing

Given that the experimental setup relies on manually layered biological samples, and although electromagnetic shielding was applied, complete isolation from the environment was not achieved. As a result, the measured S11 reflection coefficient signals inevitably contain substantial noise and high-frequency fluctuations. These artifacts can significantly impact the accuracy of feature extraction and subsequent model input. To mitigate these effects and enhance signal stability, a robust signal processing protocol was implemented to suppress interference while preserving critical signal characteristics.

A two-step signal processing approach was employed, combining low-pass filtering and Savitzky–Golay smoothing. This method was chosen for its ability to effectively reduce noise while retaining essential signal features, such as peak positions and curve shape, which are crucial for accurate tissue characterization.

In the first step, a low-pass filter was applied to attenuate high-frequency components while retaining the key low-frequency characteristics of the signal [48,49]. As shown in Equation (2), a first-order low-pass filter in the continuous-time domain can be mathematically expressed as:

(2) $$H\left(s\right)=\frac{1}{1+\frac{s}{2\pi fc}}\;$$

where H(s) is the transfer function of the filter, s is the Laplace variable, and $f_{c}$ is the cutoff frequency. This first-order low-pass filter attenuates signal components with frequencies much higher than $f_{c}$. By selecting an appropriate cutoff frequency, the filter effectively suppresses high-frequency noise in the S11 signals, enhancing signal stability and reducing interference.

Following low-pass filtering, Savitzky–Golay smoothing was applied to further reduce residual noise while maintaining key signal features [50,51]. As shown in Equation (3), this method uses a local polynomial regression to approximate the signal within a moving window, and the smoothed value ${\hat{y}}_{i}\;$at position $x_{i}$ is calculated as:

(3) $${\hat{y}}_{i}=\;\sum_{j=\;-k}^{k}C_{j}\cdot y_{i+j}\;$$

where $y_{i+j}$ are the original signal values within the window, $C_{j}\;$ are the convolution coefficients determined by polynomial fitting of degree *d*, and 2*k* + 1 is the window size. In our experiments, the S11 signals were smoothed using a filter with a window size of 23 and a third-order polynomial fitting. These parameters were chosen to balance smoothing performance and feature preservation, ensuring that critical signal characteristics such as peak positions and curve shape were retained. This approach provides a computationally efficient and effective means of smoothing noisy data while minimizing distortion.

**Figure 5:**
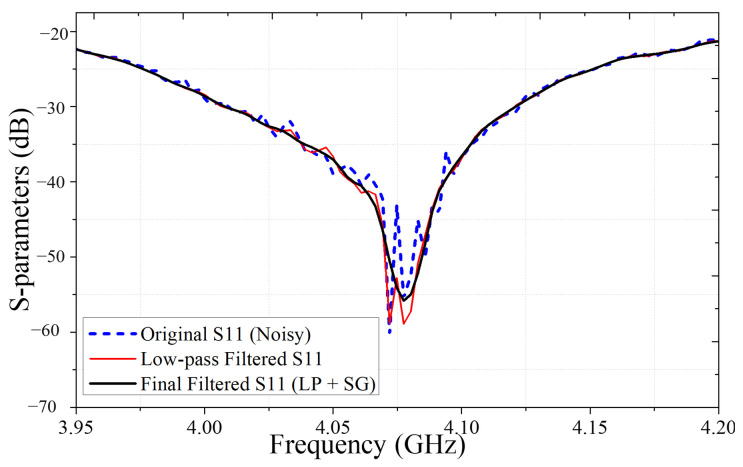
Comparison of original and filtered S-parameters (S11) signals using low-pass and Savitsky–Golay filtering.

An example of the signal processing results is shown in Figure 5, where the blue dashed line represents the raw S11 signal, the black solid line shows the result after low-pass filtering, and the red solid line indicates the final signal after both filtering and smoothing.

**Figure 6:**
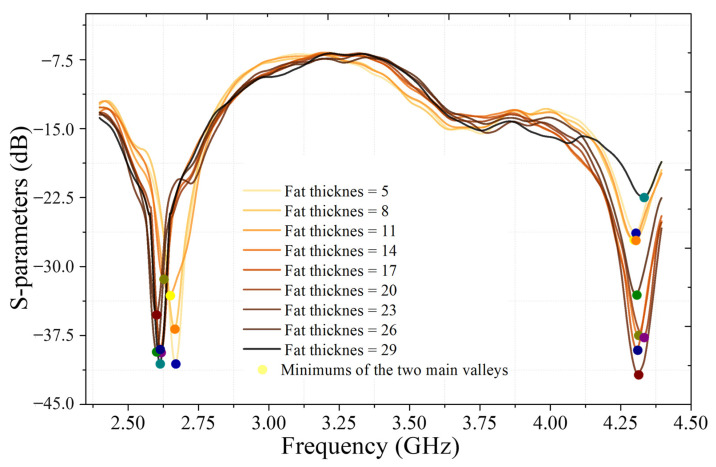
Comparison of S-parameter (S11) signals for pork samples with varying fat thickness but consistent muscle thickness (30 mm).

After preprocessing, two key types of features were extracted from each signal: resonance frequencies and signal loss. Resonance frequencies were identified as the most prominent local minima or valleys in the smoothed S11 curve, reflecting the interaction between the incident electromagnetic wave and the underlying tissue structure [47]. Signal loss was defined as the amplitude difference in decibels between the peaks and valleys surrounding the resonance region, quantifying the extent of energy absorption or reflection by the biological materials [52]. These features were selected based on prior studies demonstrating their relevance and sensitivity to variations in tissue thickness and composition. The effectiveness of this extraction approach is exemplified by the distinct differences in S11 signals corresponding to varying fat thickness at a constant muscle thickness of 30 mm, as illustrated in Figure 6. These extracted features were subsequently used as input parameters for predictive modeling, as described in the following section.

### 2.4. Statistical Analysis and Model Training

**Figure 7:**
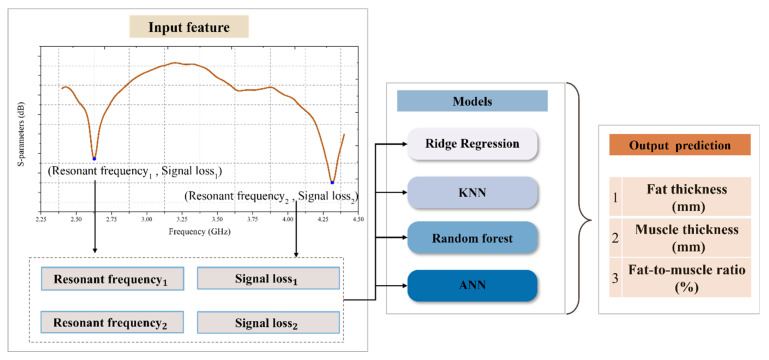
Workflow for predicting tissue composition using resonance-based features and machine learning models.

Following signal preprocessing, two sets of features were extracted for each sample: the resonance frequencies corresponding to the two dominant valleys in each S11 signal, along with their associated signal loss values. These features were selected based on their demonstrated relevance and sensitivity to variations in tissue thickness and composition, as established in prior studies. The extracted features were then used as input variables for regression model training, with the objective of predicting three target variables: fat thickness, fat-to-muscle ratio, and muscle thickness. The analysis aimed to compare the predictive performance of multiple regression models to identify the most effective modeling approach. The overall workflow of the feature extraction and prediction process is illustrated in Figure 7.

Given the limited dataset size, four regression models widely used in small-scale biomedical prediction problems were employed: Ridge Regression, K-Nearest Neighbors (KNN) Regression, Random Forest Regression, and a feedforward Artificial Neural Network (ANN) [53,54,55,56]. These models were chosen for their robustness and adaptability in handling small datasets with varying degrees of complexity. All models were trained and evaluated using the same dataset split, with 90% of the data allocated for training and 10% for testing [53]. A fixed random seed of 1 was used to ensure reproducibility. Prior to training, all input and output variables were standardized to zero mean and unit variance to enhance model performance and stability.

Ridge Regression was selected for its ability to introduce an $L_{2}$ regularization term to the ordinary least squares objective. This regularization technique reduces model variance and mitigates overfitting, particularly in the presence of collinearity or limited data. As shown in Equation (4), the Ridge loss function is defined as:

(4) $$J\left(w\right)=\;\sum_{i=1}^{n}{\left(y_{i}-w^{T}x_{i}\right)}^{2}+\alpha {\left\|\omega \right\|}^{2}$$

where $\chi_{i}\in R^{d}$ is the input feature vector, $y_{i}\in R^{3}$ is the target output vector, α=1.2 is the regularization coefficient, and w is the model weight vector. This method is particularly effective in stabilizing linear models with multiple correlated inputs, thereby enhancing prediction accuracy in small-sample scenarios.

K-Nearest Neighbors (KNN) regression was employed for its flexibility in modeling nonlinear relationships. As shown in Equation (5), the predicted output for a sample x is computed by averaging the outputs of the *k* = 3 nearest neighbors from the training set, based on Euclidean distance:

(5) $$\hat{y}\left(x\right)=\frac{1}{k}\sum_{i\in N_{k}\left(x\right)}y_{i}$$

where $N_{k}(x)$ denotes the set of k closest training samples. While KNN regression is highly adaptable, it can be sensitive to noisy inputs, necessitating careful preprocessing and validation.

Random Forest regression was implemented using a multi-output regression strategy, comprising 200 decision trees with a maximum depth of 8. Feature selection at each split was performed using the square-root heuristic, and bootstrap aggregation was enabled to enhance model robustness. A fixed random seed of 1 was used to ensure consistency. As shown in Equation (6), the final prediction is obtained by averaging the outputs of all individual trees:

(6) $$\hat{y}\left(x\right)=\frac{1}{T}\sum_{t=1}^{T}f_{t}\left(x\right)$$

where T=200 and $f_{t}$ denotes the output of the t-th decision tree. Random Forests are known for their robustness to overfitting and their ability to handle mixed-feature spaces, making them well-suited for complex biomedical datasets.

The Artificial Neural Network (ANN) employed in this study is a multilayer perceptron consisting of an input layer, two hidden layers with 32 and 16 ReLU-activated neurons, respectively, and a linear output layer corresponding to the three regression targets. As shown in Equation (7), the network’s feedforward mapping is represented as:

(7) $$\hat{y}=\;w_{3}\cdot \sigma \left(w_{2}\cdot \sigma \left(w_{1}\cdot x+b_{1}\right)+b_{2}\right)+b_{3}$$

where σ(⋅) denotes the ReLU activation function. The model was optimized using the Adam algorithm with a learning rate of 0.01, and trained to minimize the mean squared error (MSE) loss. As shown in Equation (8), the loss function is defined as:

(8) $$L=\frac{1}{n}\sum_{i=1}^{n}{\left\|{\hat{y}}_{i}-y_{i}\right\|}^{2}$$

To mitigate overfitting, early stopping was applied based on validation loss monitoring. This approach ensures that the model generalizes well to unseen data, thereby enhancing predictive accuracy in small-sample scenarios.

All models were evaluated using a consistent set of quantitative metrics to ensure a fair comparison. The Pearson correlation coefficient r was used to measure the strength of the linear relationship between predicted and true values, while the mean absolute error (MAE) was employed to assess predictive accuracy. These metrics were computed for each of the three target variables—fat thickness, fat-to-muscle ratio, and muscle thickness—allowing a comprehensive evaluation of model performance under consistent experimental conditions.

## 3. Results

### 3.1. Representative S11 Responses for Meat and Oil–Water Samples

Following MiS-based acquisition of S11 spectra under controlled, matched-layer-thickness conditions, a consistent global pattern—two pronounced resonance valleys (P1 and P2) flanking a comparatively flat mid-band—was observed across all sample categories. Within the 2.4 to 4.4 GHz window, every curve remained negative, confirming that surface reflection was dominated by impedance mismatch. Quantitative inspection of the extracted valley parameters reveals that pork muscle consistently produced the deepest minima, with P1 descending to −38.7 dB at 25 mm and P2 to −32.6 dB at 30 mm; beef muscle followed with P1 values between −37.2 dB and −27.9 dB and P2 values stabilizing near −20 dB, while the oil–water mixture exhibited the shallowest excursions, its P1 ranging from −33.1 dB to −24.9 dB and P2 from −27.5 dB to −19.9 dB, as presented in Table 1 These systematic differences in both amplitude and spectral position arise from the underlying dielectric contrast: the higher water and electrolyte content of pork engenders greater dielectric loss and thus a stronger impedance discontinuity, whereas the oil–water medium, characterized by lower permittivity and conductivity, reflects proportionally less incident energy.

Closer examination of the frequency-dependent behavior, as presented in Table 1, reveals distinct dispersion patterns in the resonant frequencies (P1 and P2) across the three sample types in response to changes in thickness. In pork muscle, the primary resonance (P1) exhibits a clear downward shift from 3.20 GHz at 25 mm to 2.63 GHz at 40 mm, while the secondary resonance (P2) simultaneously shifts upward from 3.75 GHz to 4.31 GHz. This opposing trend suggests that increasing thickness modifies the effective dielectric boundary conditions in a manner that differentially affects the two resonance modes. Beef muscle, by contrast, maintains near-constant P1 and P2 positions across all tested thicknesses, remaining centered around 3.4 GHz and 3.2 GHz, respectively. Such stability indicates a relatively homogeneous dielectric structure that is less sensitive to geometrical variation. In the case of the oil–water mixture, a more complex pattern emerges: P1 shifts markedly upward from 3.18 GHz to 4.06 GHz, while P2 moves in the opposite direction, decreasing from 3.42 GHz to 2.57 GHz. These pronounced and non-monotonic shifts reflect the highly dispersive nature of emulsified media, where small changes in thickness can lead to substantial alterations in the electromagnetic field distribution due to frequency-dependent permittivity. The comparison across sample types of thus underscores material-specific differences in dielectric dispersion and highlights the varying degrees of sensitivity to structural changes under microwave excitation.

**Figure 8:**
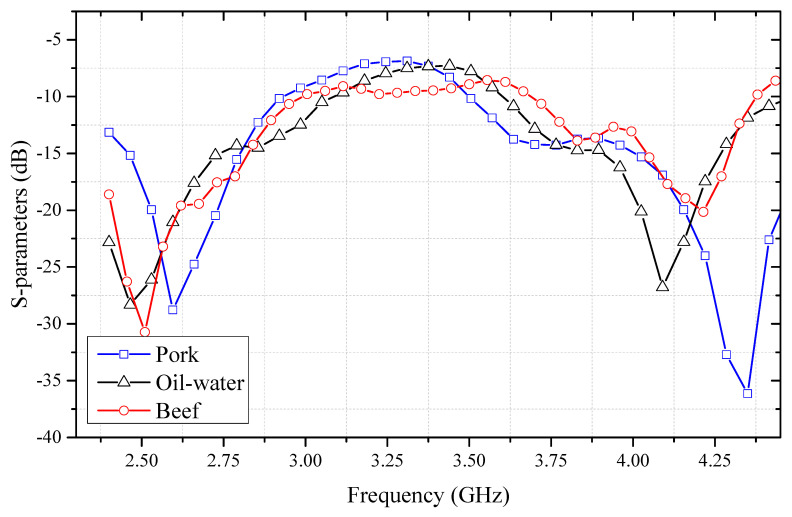
S-parameters (S11) of pork, beef, and oil–water mixture from 2.4 to 4.4 GHz.

Figure 8 summarizes representative S11 curves from pork, beef, and oil–water samples at similar thickness levels. The first resonance dip near 2.5 GHz exhibits similar S-parameter loss values across all three samples, indicating a common absorption behavior at lower frequencies. In contrast, the second resonance peak close to 4.25 GHz shows noticeable differences in peak values, particularly between the oil–water and meat-based samples, suggesting species-dependent variations in electromagnetic characteristics. Nevertheless, the overall trend of the curves remains consistent, implying that discriminative features can be systematically extracted to support predictive modeling. In addition, the beef sample exhibits additional local minima in its response curve compared to the other samples, indicating more complex electromagnetic interactions or structural heterogeneity. This variability likely arises from the heterogeneous microstructure of meat tissues such as intramuscular fat distribution and fibrous texture—which introduces subtle spatial variation in impedance and scattering. Such signal irregularities may influence the generalizability and stability of trained models and therefore need to be carefully considered during data preprocessing, feature selection, and model development.

### 3.2. Model Performance Comparison

To evaluate and compare the predictive effectiveness of different regression models across various biological materials, we trained and tested four algorithms—Ridge Regression, KNN, RF, and ANN—separately for each sample type: pork, beef, and oil–water mixtures. A total of 36 samples were used in the analysis, with 32 samples allocated for training and 4 samples reserved for testing in each case. Each model received standardized input features consisting of the two resonance frequencies and two signal loss values extracted from the preprocessed S11 spectra and was optimized using the same training/testing split and hyperparameters as previously described.

**Figure 9:**
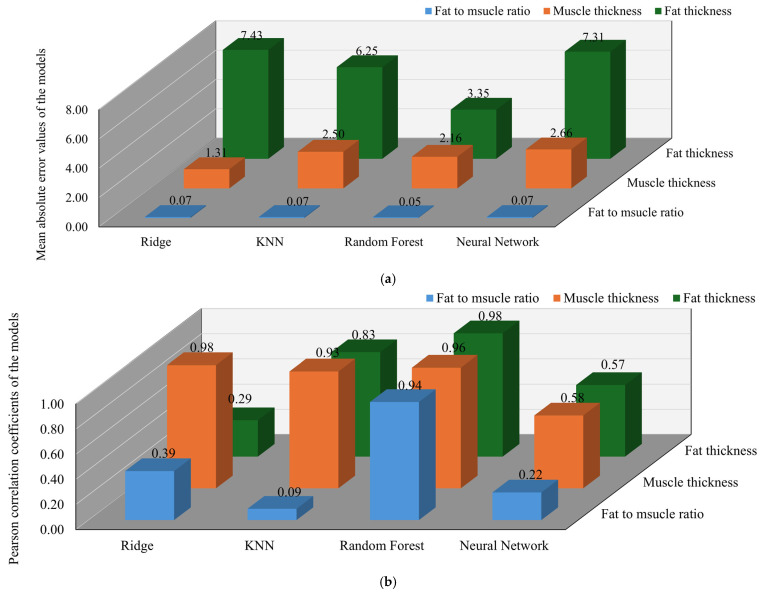
Performance evaluation of different models in predicting oil-water signal fat-to-muscle ratio, muscle thickness and fat thickness: (**a**) Comparison of mean absolute error values of the models; (**b**) Comparison of Pearson correlation coefficients of the models.

A comprehensive comparison of all models across the three target indicators reveals a slight decline in performance for the oil–water mixture samples, attributed to the relatively homogeneous dielectric structure of the medium, as shown in Figure 9. Nevertheless, the Random Forest regression model exhibited robust predictive accuracy, achieving correlation coefficients of 0.975 for fat thickness, 0.959 for the FMR, and 0.943 for muscle thickness. The corresponding MAEs were 3.34 mm, 0.071, and 2.16 mm, with percentage errors of 7.6% and 6.6% for fat and muscle thickness, respectively. In contrast, the ANN demonstrated moderate performance in fat-related predictions, with a correlation of 0.570 and an MAE of 7.77 mm for fat thickness, corresponding to a percentage error of 40.9%. For the FMR, the ANN achieved a correlation of 0.224 and an MAE of 0.059. Its prediction of muscle thickness was somewhat more accurate, with a correlation of 0.662 and an MAE of 1.24 mm, yielding a percentage error of 3.8%. The KNN regression model produced slightly weaker results, with correlation coefficients of 0.830 for fat thickness, 0.922 for the FMR, and 0.094 for muscle thickness. The associated MAEs were 6.25 mm, 0.073, and 2.50 mm, with percentage errors of 32.9% for fat thickness and 7.7% for muscle thickness. Ridge regression exhibited the poorest overall performance, particularly in fat-related predictions. For fat thickness, it yielded a correlation of 0.285 and an MAE of 7.43 mm, with a percentage error of 39.1%. For the FMR, the model achieved a correlation of 0.386 and an MAE of 0.10. Although its prediction of muscle thickness showed a high correlation of 0.978, the MAE was 3.12 mm, corresponding to a percentage error of 9.6%.

**Figure 10:**
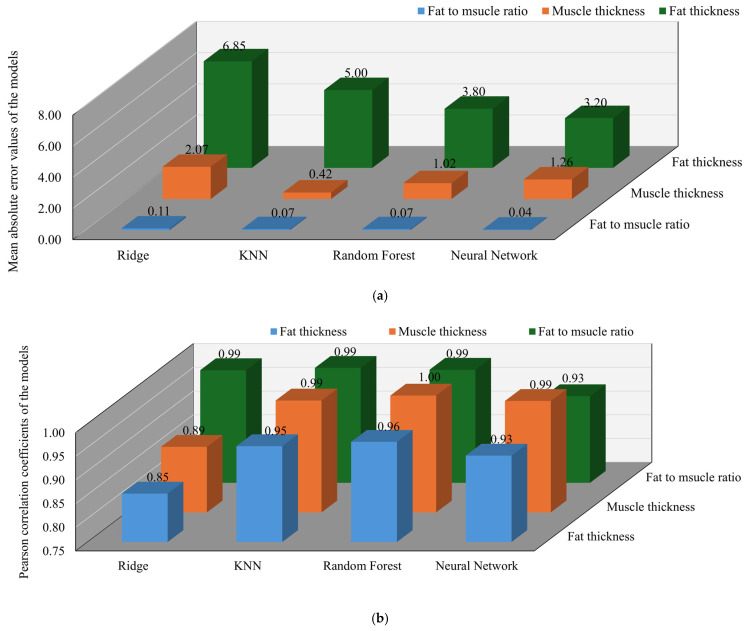
Performance evaluation of different models in predicting pork fat-to-muscle ratio, muscle thickness and fat thickness: (**a**) Comparison of mean absolute error values of the models; (**b**) Comparison of Pearson correlation coefficients of the models.

The regression performance of each model across the three prediction tasks is visually summarized in Figure 10. The Random Forest model demonstrated the highest overall accuracy for the pork dataset across all prediction targets. It achieved a correlation coefficient of 0.978 for fat thickness, with a MAE of 3.34 mm and a percentage error of 16.7%. For the fat-to-muscle ratio, the model attained a correlation of 0.943 and an MAE of 0.070. The muscle thickness prediction exhibited a correlation of 0.959 and an MAE of 2.15 mm with a percentage error of 6.4%. In contrast, the ANN showed relatively weaker results for fat-related features. For fat thickness, the ANN achieved a correlation of 0.174 and an MAE of 7.42 mm with a percentage error of 37.1%. The fat-to-muscle ratio prediction had a correlation of 0.391 and an MAE of 0.058. Similarly, the ANN’s performance in predicting muscle thickness was limited, with a correlation of 0.135 and an MAE of 3.27 mm and a percentage error of 9.7%. The KNN model yielded moderate results. For fat thickness, it attained a correlation of 0.830 and an MAE of 6.25 mm with a percentage error of 31.3%. The fat-to-muscle ratio prediction had a correlation of 0.094 and an MAE of 0.072. The muscle thickness prediction achieved a correlation of 0.927 and an MAE of 2.50 mm with a percentage error of 7.4%. As expected from a linear model, Ridge regression performed the worst in fat-related predictions. For fat thickness, it achieved a correlation of 0.285 and an MAE of 7.43 mm with a percentage error of 39.1%. The fat-to-muscle ratio prediction had a correlation of 0.386 and an MAE of 0.10. Although Ridge regression achieved a high correlation of 0.978 in predicting muscle thickness, the MAE remained relatively high at 1.31 mm with a percentage error of 3.9%.

**Figure 11:**
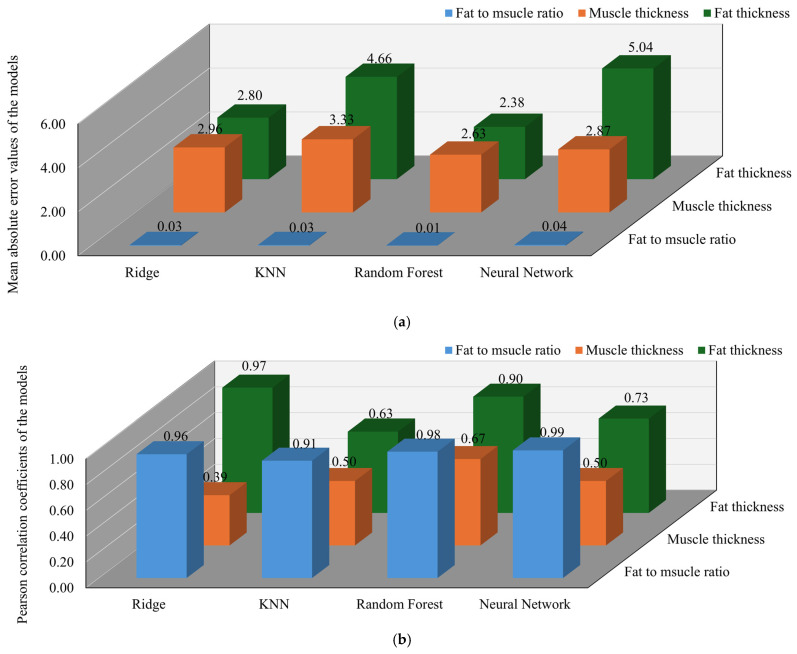
Performance evaluation of different models in predicting beef fat-to-muscle ratio muscle thickness and fat thickness: (**a**) Comparison of mean absolute error values of the models; (**b**) Comparison of Pearson correlation coefficients of the models.

The regression performance across all models and prediction targets is summarized in Figure 11. For the beef dataset, which is characterized by a more stable and uniform dielectric response, the models exhibited relatively improved and consistent performance compared to other tissue types. The Random Forest model once again demonstrated the highest accuracy, achieving correlation coefficients of 0.900 for fat thickness, 0.980 for the fat-to-muscle ratio, and 0.670 for muscle thickness. The corresponding mean absolute errors were 2.38 mm, 0.01, and 2.63 mm with percentage errors of 11.9% and 7.5% for fat and muscle thickness, respectively. The ANN also showed strong performance in fat-related predictions, with correlation values of 0.730 for fat thickness and 0.990 for the fat-to-muscle ratio. The associated mean absolute errors were 5.04 mm and 0.04 with percentage errors of 25.2% for fat thickness. However, its accuracy for muscle thickness was lower, with a correlation of 0.500 and an error of 2.87 mm with a percentage error of 8.2%. The KNN model delivered acceptable results overall, with correlation values of 0.630 for fat thickness, 0.910 for the fat-to-muscle ratio, and 0.500 for muscle thickness. The corresponding mean absolute errors were 4.66 mm, 0.03, and 3.33 mm with percentage errors of 23.3% and 9.5% for fat and muscle thickness, respectively. Ridge Regression exhibited the weakest performance in predicting muscle thickness, with a correlation of 0.390 and an error of 1.31 mm with a percentage error of 3.7%. For fat thickness and the fat-to-muscle ratio, the model achieved correlation values of 0.970 and 0.960, respectively, with corresponding errors of 2.80 mm and 0.03 and a percentage error of 14.0% for fat thickness.

Overall, the Random Forest model consistently outperformed other methods across all sample types and prediction targets, demonstrating the highest correlation coefficients and the lowest mean absolute errors. Its performance was particularly robust in both fat and muscle thickness estimation, reflecting its strong capability in handling small datasets with complex, nonlinear relationships. The artificial neural network showed competitive results in fat-related predictions, especially in the beef and oil–water datasets, but generally underperformed in muscle thickness prediction. K-nearest neighbors yielded moderate and relatively stable results, while Ridge Regression consistently demonstrated the weakest performance, especially in the presence of nonlinear features. Notably, model performance varied across sample types: pork presented greater variability and modeling difficulty, beef showed the most consistent and accurate results due to its stable dielectric properties, and the oil–water mixture, despite being more homogeneous, posed challenges in muscle-related predictions. These findings underscore the critical role of sample-specific model tuning and highlight how differences in tissue structure and dielectric behavior significantly influence signal response and prediction accuracy.

### 3.3. Prediction Visualization

**Figure 12:**
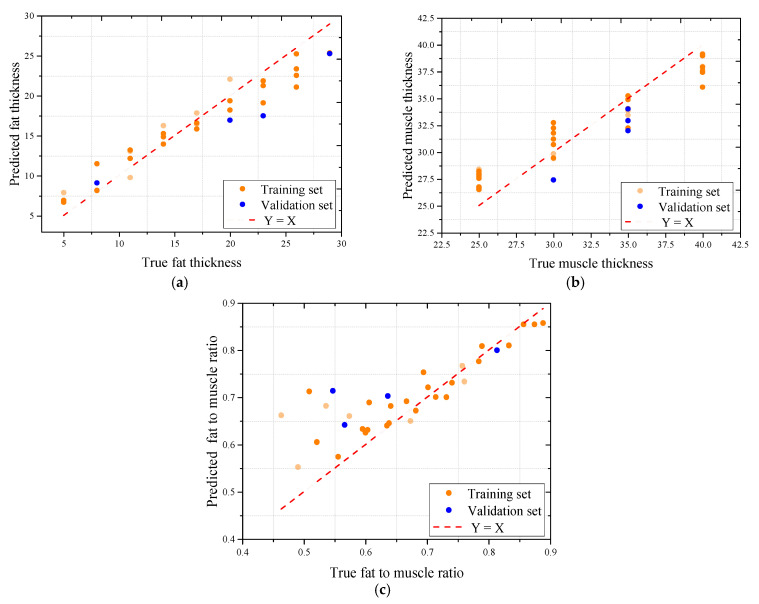
The correlation coefficient between pork signal actual and microwave predicted indicators using the Random Forest model: (**a**) The association for fat thickness; (**b**) The association for muscle thickness; (**c**) The association for fat-to-muscle ratio.

**Figure 13:**
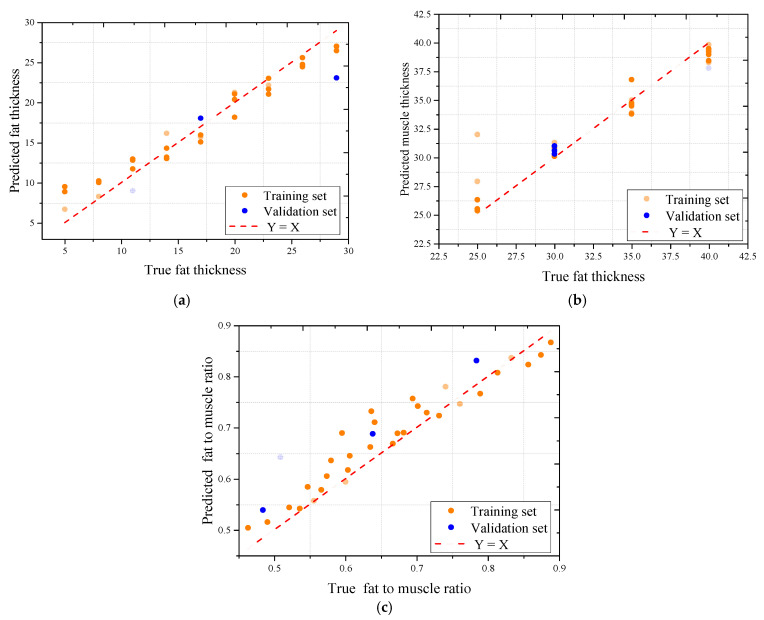
The correlation coefficient between oil-water signals actual and microwave predicted indicators using the Random Forest model: (**a**) The association for fat thickness; (**b**) The association for muscle thickness; (**c**) The association for fat-to-muscle ratio.

To further assess the predictive performance of the trained models, scatter plots were generated to compare the predicted and actual values using the best-performing model, Random Forest, across all three sample types. As shown in Figure 11, Figure 12 and Figure 13, the model demonstrated strong predictive capability for most target variables, with Pearson correlation coefficients consistently exceeding 0.95 for fat thickness and fat-to-muscle ratio in all datasets. The prediction of muscle thickness, however, exhibited more variation depending on sample type.

Among the three tested materials, the oil–water mixture achieved the highest overall prediction accuracy. As shown in Figure 12, correlation coefficients reached 0.975 for fat thickness, 0.943 for muscle thickness, and 0.959 for fat-to-muscle ratio, with minimal deviations between predicted and actual values. This strong performance is attributed to the structural homogeneity and continuous dielectric layers within the oil–water samples, which lack the internal voids and anisotropies commonly observed in biological tissues.

The pork dataset also showed high model performance, particularly in fat-related predictions. As illustrated in Figure 13, Random Forest achieved R values of 0.978 for fat thickness, 0.959 for muscle thickness, and 0.943 for fat-to-muscle ratio, with low mean absolute errors. These results suggest that although pork tissue contains some structural heterogeneity, it remains more uniform than beef in terms of microwave response.

**Figure 14:**
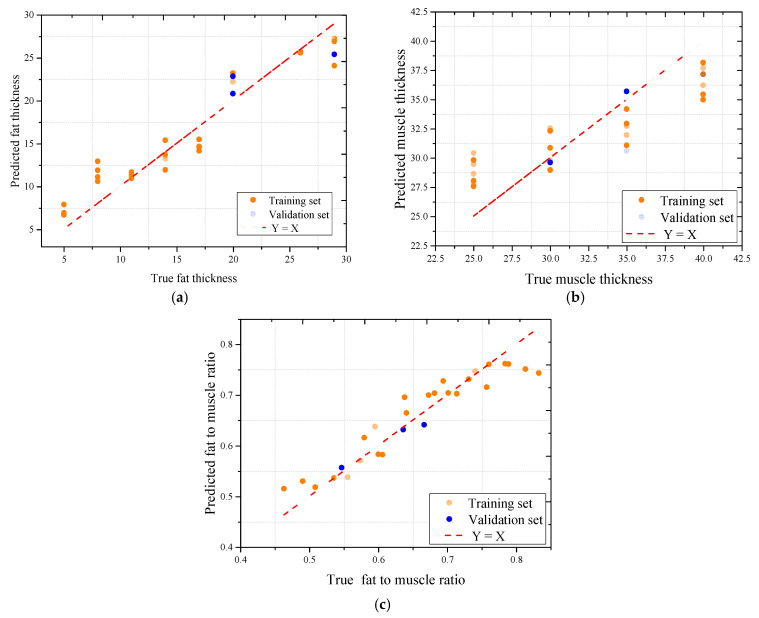
The correlation coefficient between beef signals and microwave predicted indicators using the Random Forest model: (**a**) The association for fat thickness; (**b**) The association for muscle thickness; (**c**) The association for fat-to-muscle ratio.

In contrast, the beef dataset exhibited greater variability, particularly in muscle thickness prediction. As shown in Figure 14, the correlation coefficient for muscle thickness dropped to 0.670, while the fat thickness and fat-to-muscle ratio predictions remained accurate with R values of 0.900 and 0.980, respectively. This discrepancy is likely due to the complex and irregular internal structure of shredded beef muscle, which introduces dielectric inconsistencies that hinder the model’s ability to learn consistent patterns from the S11 signal. The heterogeneous nature of beef muscle, characterized by varying fiber orientations and interstitial spaces, may contribute to the observed variability in microwave response. Additionally, the presence of fat marbling within the beef samples could further complicate the signal interpretation, leading to the reduced prediction accuracy for muscle thickness. These findings suggest that while the proposed microwave system can effectively quantify fat-related parameters, the prediction of muscle thickness in complex biological tissues such as beef may require further refinement of the model or additional preprocessing techniques to account for structural heterogeneity.

Despite these differences, the Random Forest model maintained low absolute errors across all datasets. The MAE for fat thickness predictions was around 3.0 mm, while for muscle thickness, it ranged between 2.1 and 2.6 mm depending on tissue type. For fat-to-muscle ratio, the MAE remained consistently low, as small as 0.01 in the beef dataset and below 0.7 in other cases. These results demonstrate that even under limited-sample conditions, accurate regression modeling is achievable with appropriate feature extraction and robust ensemble learning techniques.

Collectively, these results reinforce the importance of tailoring models to the specific dielectric and structural properties of each tissue type. They also highlight the ability of Random Forest regression to generalize well across biologically and physically diverse materials when supported by effective signal preprocessing and feature engineering.

### 3.4. K-Fold Cross-Validation Results

To further assess the stability of the proposed Random Forest model under the present limited-sample setting, we performed stratified 10-fold cross-validation (K = 10) on each of the three sample types. For every target variable—fat thickness, muscle thickness, and fat-to-muscle ratio—we computed r and MAE on each validation fold. The resulting Std across the ten folds are summarized in Table 2; a smaller Std denotes lower fold-to-fold variability and thus higher model stability.

Ten-fold cross-validation consistently validates the robustness of the Random Forest model across the three tested substrates, highlighting both its predictive stability and the influence of tissue structure on estimation accuracy. For the oil–water mixture, the model achieves near-ideal performance, with correlation coefficients exceeding 0.969, standard deviations below 0.04, and mean absolute errors tightly constrained around 3 mm for fat thickness and 0.034 for the fat-to-muscle ratio. These results emphasize the advantage of a homogeneous, well-layered dielectric structure, which produces stable and repeatable microwave signatures that can be modeled with high fidelity.

When applied to porcine tissue, the model maintains similarly high predictive accuracy, with mean correlations of 0.975 for fat thickness, 0.947 for the fat-to-muscle ratio, and 0.952 for muscle thickness. Standard deviations of 0.027, 0.024, and 0.035, respectively, further underscore the reliability of these predictions. This suggests that even naturally heterogeneous substrates, such as pork, which retains a comparatively uniform anatomical layering, can be quantified with excellent consistency.

In contrast, results from bovine tissue reveal both the strengths and limitations of the current framework. While predictive accuracy remains strong for fat-related variables, with correlation coefficients of 0.930 for fat thickness and 0.936 for the fat-to-muscle ratio, the estimation of muscle thickness shows a marked decline, with correlation dropping to 0.697 ± 0.061 and the largest observed error variability. This performance degradation reflects the inherently complex microstructure of beef muscle, characterized by irregular intramuscular fat distribution, nonuniform fiber orientation, and higher dielectric variability, all of which complicate the modeling process.

These findings demonstrate that the proposed microwave-based framework achieves stable and high-fidelity predictions for fat thickness and fat-to-muscle ratio across both controlled and biological substrates, thereby confirming its utility for body composition assessment. However, the reduced performance observed in muscle-thickness estimation within bovine tissue underscores the critical impact of structural heterogeneity on model stability. Addressing this limitation—through improved signal processing, multimodal data fusion, or refined anatomical priors—represents an important direction for future research. Ultimately, these results establish a foundation for extending the methodology toward reliable in vivo applications, where variability in tissue structure will be an unavoidable challenge.

## 4. Discussion

This study experimentally validated the feasibility of using the portable MiS to estimate the composition of biological tissues by analyzing the S11 reflection coefficient. The results demonstrated that key features extracted from the preprocessed S11 signals, namely resonance frequencies and signal loss, could effectively predict fat thickness, fat-to-muscle ratio, and muscle thickness across various biological samples. Among the four regression models evaluated, the Random Forest algorithm consistently exhibited the highest prediction accuracy and robustness for pork, beef, and oil–water mixtures. These findings not only confirmed the advantage of Random Forest in handling complex biological data but also highlighted its general applicability across different sample types.

A notable observation was that the oil–water mixture samples yielded the most consistent and accurate predictions, which can be attributed to the homogeneity of their dielectric properties. Unlike biological tissues, the oil and water layers form smooth, continuous interfaces that support stable electromagnetic wave propagation with minimal signal distortion. In contrast, pork and beef samples introduced increased variability due to their complex internal architectures, heterogeneous moisture distribution, and structural irregularities. These factors resulted in more erratic reflection patterns, posing greater challenges to model generalization. Between the two meat types, pork samples achieved higher prediction accuracy than beef in terms of both correlation and mean absolute error. This discrepancy is likely due to differences in tissue density, fiber alignment, and fat distribution. The relatively uniform dielectric properties of pork, particularly in the fat layers, may have facilitated more reproducible microwave responses. In contrast, beef tissues, with more fragmented muscle structures, exhibited greater signal fluctuation and predicted uncertainty.

In addition to demonstrating strong predictive performance, the MiS system offers significant practical advantages. Its compact and lightweight design, integrated with essential radio-frequency components and Bluetooth telemetry, enables rapid in-field measurements without the need for bulky instrumentation. The system’s low cost and ability to adapt to various sample types using a unified hardware configuration make it an attractive solution for scalable deployment in agri-food quality control, veterinary diagnostics, and biomedical sensing. Overall, the findings highlight the MiS platform as a promising tool for accurate, non-destructive tissue composition analysis across a range of biological materials.

Despite the promising results, this study has several limitations. First, the dataset size was relatively small. In particular, multiple repeated entries for muscle thickness were intentionally included to capture the variability in fat thickness under different muscle thicknesses. This repetition may have affected the prediction performance and model fitting for muscle thickness, potentially reducing accuracy. Furthermore, due to the difficulty in preparing samples with fine step-size variations and the use of 1 mm as the minimum resolution for ground truth measurements, the generalizability of the trained models to a broader range of tissue types or in vivo conditions remains limited. Future applications of the MiS in more extensive experimental scenarios, accompanied by more precise data acquisition and measurement, are expected to address these limitations and enhance the accuracy and reliability of the system.

Additionally, the process of grinding and reassembling meat layers could have introduced geometric inconsistencies and discontinuities between tissue layers, potentially impacting the repeatability of results. The reduced prediction performance in the beef muscle group is likely due to the reconstitution of minced beef, which presented challenges in maintaining a consistent fat layer under experimental conditions. In later stages of the experiment, the fat component of beef partially melted at room temperature, affecting signal detection and increasing data dispersion [57]. In contrast, no such degradation was observed during pork sample preparation, and its detection and training performance remained stable. These findings suggest that structural complexity and lower cohesion of beef fat tissues may have contributed to the observed discrepancies. This highlights the need for improved sample preparation protocols in future studies to minimize variability introduced by material inconsistencies.

Moreover, the current system has not yet been tested or deployed in real-world environments. For large-scale production and practical use, design considerations such as device weight and size must be addressed. Future improvements should focus on implementing a PCB-based solution that integrates all components into a compact and lightweight board, maintaining or even enhancing current system performance. Nonetheless, this study lays a practical foundation for noninvasive tissue characterization using compact microwave sensors. The demonstrated ability to extract tissue composition and thick information from S11 signals with reasonable accuracy provides a viable pathway for future integration into portable diagnostic systems. Simultaneously, there is significant potential to extend the MiS system’s application to the field of pet body composition analysis. With the increasing demand for accurate, noninvasive health monitoring tools in veterinary care, a portable microwave-based device capable of assessing fat, muscle, and overall body composition could revolutionize routine animal health assessments. This would facilitate early detection of obesity, malnutrition, or muscle wasting conditions in pets, supporting more personalized and timely interventions. Moreover, the adaptability of the system to different species and tissue types of positions as a versatile tool for broader animal health management beyond companion animals, potentially benefiting livestock and wildlife monitoring as well. Future work will focus on expanding the diversity of samples to encompass a wider range of species and physiological conditions, refining feature extraction and selection methods, and integrating advanced machine learning techniques—including deep learning—to enhance model accuracy, robustness, and generalizability. Through these continued advancements, the MiS system is expected to play a pivotal role in both human and veterinary biomedical fields, providing an efficient, accurate, and user-friendly solution for noninvasive tissue characterization across diverse applications.

## 5. Conclusions

In this study, we developed a compact ultra-wideband microwave system capable of rapid, non-invasive prediction of fat thickness, muscle thickness, and fat-to-muscle ratio in ex vivo pork, beef, and oil–water samples. By leveraging the sensitivity of the S11 reflection coefficient to dielectric heterogeneity and incorporating advanced signal processing with machine learning algorithms, the proposed system demonstrated high prediction accuracy and reliable generalization across distinct biological and synthetic materials.

Two key innovations contributed to the system’s performance:

1. The handheld MiS system operates over a frequency range from 2.4 to 4.4 GHz and integrates all essential RF components into a portable design. It provides S11 measurements with functionality similar to that of commercial VNAs, along with real-time Bluetooth transmission. A two-stage signal preprocessing approach, including low-pass filtering and Savitzky–Golay smoothing, effectively reduced noise while preserving the critical resonance and attenuation features in the spectra.
2. Among the evaluated models, Random Forest consistently achieved the highest prediction accuracy across all targets and sample types. It reached correlation coefficients up to 0.978 and maintained mean absolute errors below 3.5 mm for fat thickness and under 1.0 for fat-to-muscle ratio. Notably, it preserved reliable performance on both structurally homogeneous samples (oil–water mixture) and highly heterogeneous meat tissues (pork and beef). In contrast, linear Ridge Regression and neural networks struggled with nonlinear and structurally variable samples, particularly in muscle thickness estimation.

Furthermore, comparative analysis revealed that sample-specific factors such as tissue microstructure and dielectric uniformity significantly influenced prediction performance. The oil–water mixture yielded the most accurate and consistent results due to its continuous and layered dielectric properties. Pork samples exhibited moderate variability but outperformed beef, which showed the greatest signal fluctuation and lowest muscle prediction accurately due to the inhomogeneous nature of shredded muscle tissue.

These findings demonstrate that with appropriate signal preprocessing and model selection, accurate tissue composition estimation from S11 microwave signals is feasible even under limited-sample conditions. The proposed MiS platform holds promise for low-cost, portable applications in agri-food quality control, veterinary diagnostics, and non-destructive biomedical sensing.

## Figures and Tables

**Table 1:** Statistical features of resonant frequency and signal loss at two dominant valleys (P1 and P2) for different samples and muscle thicknesses.

Sample	Muscle thickness (mm)	Mean Freq (GHz)		Mean S11(dB)		Std Freq (GHz)		Std S11 (dB)	
		P1	P2	P1	P2	P1	P2	P1	P2
Oil-Water	25	3.18	3.42	−33.10	−27.52	0.85	0.86	5.85	2.78
	30	2.56	4.12	−26.43	−19.86	0.01	0.04	3.04	0.78
	35	2.89	3.74	−24.97	−19.99	0.67	0.66	1.94	2.56
	40	4.06	2.57	−27.80	−23.46	0.02	0.02	3.48	1.44
Pork	25	3.20	3.75	−38.69	−31.07	0.84	0.85	2.58	5.65
	30	3.39	3.56	−37.93	−32.59	0.90	0.90	7.87	5.74
	35	2.82	4.12	−34.25	−29.36	0.55	0.56	4.22	3.85
	40	2.63	4.31	−36.27	−26.54	0.02	0.02	3.40	3.87
Beef	25	3.43	3.22	−31.98	−22.02	0.85	0.88	4.86	2.47
	30	3.42	3.20	−37.17	−20.02	0.83	0.87	6.64	2.39
	35	3.42	3.21	−32.86	−20.01	0.86	0.87	6.70	2.76
	40	3.39	3.20	−27.95	−20.37	0.82	0.86	4.29	2.59

**Table 2:** Predictive Performance under Ten-Fold Cross-Validation (Mean ± Std).

Sample	Target Variable	Correlation		MAE	
		Mean	Std	Mean	Std
Oil-Water	Fat thickness	0.976	0.029	2.839	0.788
	Fat-to-muscle ratio	0.978	0.025	0.034	0.018
	Muscle thickness	0.969	0.039	1.566	0.642
Pork	Fat thickness	0.975	0.027	3.366	0.446
	Fat-to-muscle ratio	0.947	0.024	0.027	0.025
	Muscle thickness	0.952	0.035	2.122	0.693
Beef	Fat thickness	0.930	0.056	2.126	0.828
	Fat-to-muscle ratio	0.936	0.034	0.028	0.008
	Muscle thickness	0.697	0.061	2.746	0.817

## Data Availability

The original contributions presented in this study are included in the article. Further inquiries can be directed to the corresponding author.
